# Characterizing HIV seroconversions among a cohort of oral PrEP users in South Africa

**DOI:** 10.1002/jia2.26421

**Published:** 2025-02-22

**Authors:** Catherine E. Martin, Hlologelo Ramatsoma, Glory Chidumwa, Laura Ashleigh Cox, Saiqa Mullick

**Affiliations:** ^1^ Wits RHI and School of Public Health Affiliations for Glory Chidumwa University of the Witwatersrand Johannesburg South Africa; ^2^ Division of Epidemiology and Biostatistics Wits RHI and School of Public Health Affiliations for Glory Chidumwa University of the Witwatersrand Johannesburg South Africa

**Keywords:** adolescent girls and young women, HIV prevention, HIV testing, implementation study, PrEP, seroconversion

## Abstract

**Introduction:**

There has been significant progress in the rollout of oral pre‐exposure prophylaxis (PrEP) for the prevention of HIV. The introduction of long‐acting prevention methods holds the potential to improve HIV prevention uptake and use, however, presents unique complexities regarding HIV diagnosis and potential for resistance. Quantifying and understanding the scenarios within which seroconversions occur may help to inform approaches to identifying acute HIV in programmes delivering PrEP at scale.

**Methods:**

This paper documents ctra series of seroconversions within a large implementation study conducted in eight Department of Health facilities and four linked mobile clinics in four areas of South Africa. Using routinely collected data, we conducted a descriptive analysis of clients who seroconverted after initiating oral PrEP and determined the distribution of time from oral PrEP initiation to seroconversion as well as the proportion of days covered by oral PrEP. A seroconversion was defined as any HIV‐positive diagnosis after initiation of PrEP. Time to seroconversion was calculated as the number of days between the first PrEP initiation and the date of HIV diagnosis. The proportion of days covered by PrEP was calculated as the number of days of PrEP prescribed over the number of days between PrEP initiation and HIV seroconversion. We conducted a logistic regression to determine factors associated with seroconversion.

**Results:**

Of the 11,882 clients initiated on PrEP between January 2019 and October 2022 who attended at least one follow‐up visit, 112 (0.9%) seroconverted after PrEP initiation. Among those who seroconverted, the median proportion of days covered by PrEP between initiation and seroconversion was 33%. In the period between PrEP initiation and seroconversion, almost all (*n* = 93, 83.0%) had not used PrEP consistently, with only 19 (17.0%) having consistent PrEP use, all of whom were identified at the 1‐month follow‐up visit and were likely missed acute acquisitions. Younger age and geographical area were associated with seroconversion.

**Conclusions:**

This study reports a low number of seroconversions among a large cohort of PrEP users in a real‐world implementation study, the majority of which occurred among clients who had interrupted or discontinued PrEP use.

## 1 INTRODUCTION

There has been significant progress in the rollout of oral pre‐exposure prophylaxis (PrEP) for the prevention of HIV. However, daily pill taking as well as individual and health system barriers are challenges to ongoing effective use [1, 2, 3, 4]. The introduction of long‐acting PrEP methods, such as the dapivirine vaginal ring, injectable cabotegravir (CAB‐LA) and injectable lenacapavir, will increase options and improve choice, holding the potential to overcome some of the challenges with daily oral PrEP [5, 6] and improve overall uptake and effective use of PrEP [7].

The risk of HIV drug resistance from PrEP use is minimal compared to treatment with antiretroviral therapy (ART), with actual implications on future treatment options and onward transmission largely unknown [8, 9]. The greatest potential for drug resistance among PrEP users is when initiating or using PrEP during undiagnosed acute HIV [8, 10]. Newer, long‐acting PrEP methods may delay the time to seroconversion due to viral suppression and reduced antibody response, with the potential for ongoing development of resistance mutations [11]. A noted limitation of current HIV testing strategies is that antibody‐based testing (particularly third‐generation tests) may miss acute HIV. Diagnosis of acute HIV in people using long‐acting PrEP, such as CAB‐LA, could be particularly challenging, although may be improved through the use of HIV RNA assay tests [6]. However, these tests are not without challenges. Single RNA tests have been associated with false‐positive results and may perform poorly in detecting HIV among people using CAB‐LA [12]. They are also expensive with long turnaround times, and would not be feasible or affordable in low‐ and middle‐income countries [6]. While these concerns should not hamper PrEP introduction, quantifying and understanding scenarios within which HIV acquisitions among PrEP users may occur, may inform approaches to identifying acute HIV in programmes delivering PrEP at scale.

This paper documents a series of HIV seroconversions within an oral PrEP implementation study in South Africa.

## 2 METHODS

This study is embedded within a large, real‐world implementation study, which supported the introduction of oral PrEP in South Africa, focusing on girls and young women. It is implemented within eight Department of Health facilities and four linked mobile clinics in four areas of South Africa: Tshwane in Gauteng, Gqeberha and Mthatha in the Eastern Cape and eThekwini in Kwa Zulu Natal. The project approach has been described elsewhere [13].

### 2.1 Study population and data collection

Routine data were collected from all clients ≥15 years of age accessing services at project sites at each visit and extracted from the monitoring database for the purpose of this analysis. Inclusion criteria were having initiated oral PrEP between January 2019 and October 2022 and having attended at least one clinical follow‐up visit with an HIV test conducted after initiation of PrEP. HIV testing was conducted using a rapid, point‐of‐care (POC) antibody‐based test, in line with national guidelines [14]. Test kits varied by site, depending on local procurement, and included One Step Anti‐HIV (1&2) for screening, confirmed with Colloidal Gold or Toyo Anti‐HIV 1/2 for screening, confirmed with First Response HIV 1–2.0. One Step, Colloidal Gold and First Response tests are WHO pre‐qualified; guidelines for quality assurance are detailed in national policy [14]. Any positive rapid test was confirmed using a second, confirmatory POC antibody test. Oral PrEP was provided according to national guidelines, with follow‐up 1‐month after PrEP initiation or re‐start and thereafter 3‐monthly [15]. Screening for acute HIV was conducted according to guidelines through an enquiry about acute viral symptoms such as malaise, myalgia, sore throat and rash, and examination for clinical signs including lymphadenopathy, fever, pharyngitis and rash among others [15]. If present, PrEP initiation was postponed until symptoms subsided, and a repeat HIV test 4 weeks later remained negative. Standard of care support for PrEP use was provided to all clients, including counselling on combination HIV prevention, the use of condoms, HIV vulnerability assessment and reduction, consistent use of daily oral PrEP during periods of potential exposure and information on how to correctly stop PrEP to ensure effectiveness [15]. This comprised counselling on the use of PrEP for 7 days prior to reaching full protection, with additional precautions recommended during this time, and for 28 days after the last potential exposure [15]. This was updated to recommend PrEP use for 7 days after the last exposure with revised guidelines in 2021 [16].

### 2.2 Measures and data analysis

Demographic and clinical data, including HIV test results, initiation of oral PrEP (documented script for oral PrEP) and months of PrEP prescribed, were recorded by the consulting healthcare worker. A seroconversion was defined as an HIV‐positive diagnosis after initiation of oral PrEP. Time to seroconversion was the number of days between the first PrEP initiation and the date of HIV diagnosis. A clinic visit was “on‐time” if it occurred ≤28 days after a 1‐month prescription of PrEP (each oral PrEP bottle dispensed contains 28 pills), ≤56 days after a 2‐month prescription or ≤84 days after a 3‐month prescription. Clients were categorized as having consistent PrEP use if all follow‐up clinic visits were on time. The proportion of days covered by PrEP (PrEP coverage) was calculated as the number of days of PrEP prescribed over the number of days between PrEP initiation and HIV seroconversion. In instances where the amount of PrEP prescribed was greater than the number of days between initiation and seroconversion, coverage may have been calculated at >100%. PrEP restarts were defined as PrEP prescriptions issued >28 days after the expected follow‐up date. Seroconversions were classified as a potential missed early acquisition if they occurred within an on‐time 1‐month follow‐up visit after PrEP initiation or restart. We conducted a descriptive analysis of clients who seroconverted after oral PrEP initiation and determined the distribution of time from oral PrEP initiation to seroconversion and the proportion of days covered by oral PrEP. Factors associated with seroconversion were determined using firth logistic regression, reporting odds ratios (ORs) and 95% confidence intervals (CIs). Firth logistic regression is a penalized logistic regression which allows for convergence to finite estimates for rare conditions [17, 18].

### 2.3 Ethical considerations

The study was approved by the Human Research Ethics Committees at the University of the Witwatersrand (M180806) and the World Health Organization (Wits‐PrEP‐AGYW). Approvals provided for collection and analysis of routine data, without a requirement for individual informed consent. All clients who seroconverted were initiated or referred for immediate initiation of ART, in line with national guidelines. ART is initiated on the same day, based on the results of a confirmatory rapid HIV test, without a requirement for additional HIV or drug resistance testing.

## 3 RESULTS

There were 35,375 clients (30,367 [85.8%] female, 4987 [14.1%] male and 21 [0.1%] transgender) who accessed services between January 2019 and October 2022, of whom 28,903 (81.7%) initiated oral PrEP. Of those initiated on oral PrEP, 11,882/28,903 (41.1%) attended at least one follow‐up clinical visit with a documented HIV status, and 112/11,882 (0.9%) tested HIV positive or seroconverted at any subsequent visit to the site after PrEP initiation. Baseline characteristics of clients who attended at least one follow‐up clinic visit are presented in Table 1.

**Table 1:** Demographic and socio‐behavioural characteristics of clients who attended at least one follow‐up visit after initiation on oral PrEP, by seroconversion status (*N* = 11,882)

Variable	Non‐seroconverters *N* = 11,770 (99.1%)	Seroconverters *N* = 112 (0.9%)	Total *N* = 11,882 (100%)
**Age group**			
15–17 years	1976 (16.8%)	24 (21.4%)	2000 (16.8%)
18–24 years	6354 (54.0%)	67 (59.8%)	6421 (54.0%)
25 years and older	3319 (28.2%)	21 (18.8%)	3340 (28.1%)
Missing	121 (1.0%)	0 (0.0%)	121 (1.0%)
**Gender**			
Male	2003 (17.0%)	21 (18.8%)	2024 (17.0%)
Female	9634 (81.9%)	91 (81.2%)	9725 (81.8%)
Other	133 (1.1%)	0 (0.0%)	133 (1.1%)
**Geographical area**			
Gqeberha	2211 (18.8%)	35 (31.2%)	2246 (18.9%)
Mthatha	3832 (32.6%)	40 (35.7%)	3872 (32.6%)
Kwa‐Zulu Natal	2890 (24.6%)	21 (18.8%)	2911 (24.5%)
Tshwane	2716 (23.1%)	16 (14.3%)	2732 (23.0%)
Missing	121 (1.0%)	0 (0.0%)	121 (1.0%)
**Relationship status**			
Single	1880 (16.0%)	21 (18.8%)	1901 (16.0%)
In a relationship	9239 (78.5%)	84 (75.0%)	9323 (78.5%)
Casual/open relationships	485 (4.1%)	7 (6.2%)	492 (4.1%)
Missing	166 (1.4%)	0 (0.0%)	166 (1.4%)
**Main partner's HIV status** ^a^			
Known negative	3838 (41.5%)	35 (41.7%)	3873 (41.5%)
Known positive	977 (10.6%)	6 (7.1%)	983 (10.5%)
Unknown	4424 (47.9%)	43 (51.2%)	4467 (47.9%)
**Always uses condoms**			
Yes	2605 (22.1%)	27 (24.1%)	2632 (22.2%)
No	1360 (11.6%)	12 (10.7%)	1372 (11.5%)
Sometimes	7121 (60.5%)	67 (59.8%)	7188 (60.5%)
Missing	684 (5.8%)	6 (5.4%)	690 (5.8%)
**History of an STI**			
No	10,987 (93.3%)	106 (94.6%)	11,093 (93.4%)
Yes	418 (3.6%)	4 (3.6%)	422 (3.6%)
Missing	365 (3.1%)	2 (1.8%)	367 (3.1%)
**Any STI signs and symptoms**			
No	10,862 (92.3%)	102 (91.1%)	10,964 (92.3%)
Yes	908 (7.7%)	10 (8.9%)	918 (7.7%)
**Transactional sex**			
No	11,164 (94.9%)	108 (96.4%)	11,272 (94.9%)
Yes	237 (2.0%)	2 (1.8%)	239 (2.0%)
Missing	369 (3.1%)	2 (1.8%)	371 (3.1%)
**IPV or GBV**			
No	11,150 (94.7%)	109 (97.3%)	11,259 (94.8%)
Yes	253 (2.1%)	1 (0.9%)	254 (2.1%)
Missing	367 (3.1%)	2 (1.8%)	369 (3.1%)
**Sex under the influence of alcohol or drugs** ^b^			
No	10,873 (92.4%)	103 (92.0%)	10,976 (92.4%)
Yes	531 (4.5%)	7 (6.2%)	538 (4.5%)
Missing	366 (3.1%)	2 (1.8%)	368 (3.1%)
**Injectable drug use with shared needles and/or syringes**			
Yes	232 (2.0%)	2 (1.8%)	234 (2.0%)
No	11,173 (94.9%)	108 (96.4%)	11,281 (94.9%)
Missing	365 (3.1%)	2 (1.8%)	367 (3.1%)
**Consistent PrEP use**			
Yes	1901 (16.2%)	19 (17.0%)	1920 (16.2%)
No	9869 (83.8%)	93 (83.0%)	9962 (83.8%)

Abbreviations: GBV, gender‐based violence; IPV, intimate partner violence; STI, sexually transmitted infection.

^a^ Among those in a relationship (*N* = 9323).

^b^ In the last 3 months.

The median number of weeks between PrEP initiation and seroconversion was 25.5 (minimum of 3.6 and a maximum of 184.9 weeks [about 3.5 years]). At the time of seroconversion, among the 112 individuals who seroconverted, 85 (75.9%) had not picked up PrEP on time, while 27 (24.1%) were on time. Between PrEP initiation and seroconversion, almost all who seroconverted (*n* = 93, 83.0%) had not used PrEP consistently, with 19 (17.0%) having consistent PrEP use prior to seroconversion, all of whom were identified at the 1‐month follow‐up visit and were likely missed acute acquisitions. Among individuals who seroconverted, the median proportion of days covered between PrEP initiation and seroconversion was 33%, with a minimum of 3.5% and a maximum of 112%. When excluding individuals who seroconverted at month 1 follow‐up visit (*n* = 19), PrEP coverage ranged from 3.5% to 96.5%. Excluding seroconversions identified at month 1 follow‐up (*n* = 19), 66.7% (*n* = 62/93) had restarted PrEP once and 31 individuals (33.3%) had restarted PrEP more than once prior to seroconversion. Four seroconversions occurred within 1 month of a PrEP restart. In total, there were an estimated 23 (20.5%; 23/112) individuals (19 after the first PrEP initiation and 4 after PrEP restart) who may have been missed early HIV acquisitions.

In the regression analysis, only age and geographical area were associated with seroconversion. Participants aged 18−24 (OR = 0.87; 95% CI: 0.54−1.38) and ≥25 (OR = 0.54; 95% CI: 0.30−0.96) were 13% and 46% less likely to seroconvert compared to those aged 15−17, respectively. Participants from Mthatha (OR = 0.65; 95% CI: 0.41−1.03), KZN (OR = 0.52; 95% CI: 0.30−0.89) and Tshwane (OR = 0.37; 95% CI: 0.21−0.67) were 35%, 48% and 63% less likely to seroconvert compared to those from Gqeberha, respectively (Table 2).

**Table 2:** Baseline factors associated with seroconversion, among clients who attended at least one follow‐up visit after initiation on oral PrEP (*N* = 11,882)

Variable Description	Odd ratio (95% CI)	*p*‐value
**Age group**		
15−17 years	Reference	
18−24 years	0.86 (0.54−1.36)	0.516
25 years and older	0.52 (0.29−0.94)	0.029
**Gender**		
Male	Reference	
Female	0.87 (0.54−1.40)	0.566
**Geographical area** ^a^		
Gqeberha	Reference	
Mthatha	0.65 (0.41−1.03)	0.064
Kwa‐Zulu Natal	0.52 (0.30−0.89)	0.018
Tshwane	0.37 (0.21−0.67)	0.001
**Relationship status**		
Single	Reference	
In a relationship	0.80 (0.50−1.29)	0.357
Casual/open relationships	1.35 (0.58−3.12)	0.482
**Main partner's HIV status**		
Known negative	Reference	
Known positive	0.69 (0.30−1.58)	0.379
Unknown	1.09 (0.71−1.68)	0.687
**Always uses condoms**		
No	Reference	
Yes	1.15 (0.59−2.25)	0.682
Sometimes	1.03 (0.56−1.89)	0.916
**History of an STI**		
No	Reference	
Yes	1.14 (0.44−2.95)	0.782
**Any STI signs and symptoms**		
No	Reference	
Yes	1.22 (0.65−2.32)	0.534
**Transactional sex**		
No	Reference	
Yes	1.14 (0.32−4.01)	0.842
**IPV or GBV**		
No	Reference	
Yes	0.63 (0.13−3.17)	0.576
**Sex under the influence of alcohol or drugs** ^b^		
No	Reference	
Yes	1.51 (0.72−3.20)	0.275
**Injectable drug use with shared needles and/or syringes**		
No	Reference	
Yes	1.16 (0.33−4.08)	0.820
**Consistent PrEP use**		
No	Reference	
Yes	1.08 (0.66−1.76)	0.757

Abbreviations: GBV, gender‐based violence; IPV, intimate partner violence; STI, sexually transmitted infection.

^a^ Analysis adjusted for age, Crude ORs (95% CIs) Mthatha: 0.66 (0.42−1.04), KZN: 0.46 (0.27−0.79) and Tshwane: 0.38 (0.21−0.68).

^b^ In the last 3 months.

## 4 DISCUSSION

This study describes a series of HIV seroconversions among a cohort of clients accessing oral PrEP services within routine primary care settings in South Africa. To our knowledge, this is the largest case series of seroconversions from a single real‐world PrEP programme in the region. Overall, the proportion of clients with incident HIV diagnosed after PrEP initiation was very low (<1%), consistent with low HIV incidence reported from other PrEP programmes among young women in the region [3, 19].

In this study, most seroconversions occurred in clients who had inconsistent or late PrEP pick‐ups, or a low coverage of PrEP for the period between PrEP initiation and seroconversion. This is in keeping with other implementation studies, which have reported inconsistent PrEP use among participants who seroconverted [3, 19]. Although no association was found between consistent PrEP use and seroconversion, and reasons for PrEP interruption were not explored in this analysis, it is plausible that clients who remained HIV negative were using PrEP cyclically, during periods of exposure, in keeping with the concept of prevention‐effective use [20]. It is estimated that four or more doses per week of oral PrEP in cisgender women would provide >90% oral PrEP efficacy [21]. Information and messaging around prevention‐effective use and support for cycling on and off PrEP should remain a key component of PrEP programming. The association between seroconversion and age and geographical area highlights the ongoing vulnerability of adolescents and young adults, and the geographical differences in HIV incidence.

There were few clients among whom it was plausible that acute HIV was missed at the time of PrEP initiation. The population‐based SEARCH study in rural Kenya and Uganda reported 3/25 (12%) incident HIV acquisitions at the week 4 visit after PrEP initiation [19], in keeping with the data reported in this series. The low proportion of missed acute HIV acquisitions among clients returning 1 month after PrEP initiation provides reassurance to programmes preparing to deliver long‐acting PrEP. However, further research quantifying and determining the individual and public health implications of missed acute HIV acquisitions is needed from studies delivering these methods in real‐world settings. In addition, studies evaluating testing approaches such as the use of third‐ or fourth‐generation, RNA‐based, affordable POC and/or self‐tests will be critical to optimizing testing approaches alongside new PrEP method introduction. Efforts to optimize and sustain the quality of POC HIV testing, to minimize false negative tests and maintain diagnostic accuracy must remain an important component of HIV programmes. This is especially important for PrEP programmes, given that the currently recommended HIV test algorithm prior to PrEP initiation and continued use remains a single POC rapid test [16, 22]. In addition, while it may not impact early detection of acute HIV, maintaining diagnostic accuracy of rapid tests remains critical to ensure any seroconversions are accurately identified. In 2019, the WHO recommended moving to a 3‐test algorithm for HIV diagnosis to maintain accuracy and prevent misdiagnosis [23], highlighting an immediate opportunity for improving the quality of HIV testing services.

Further to this, monitoring and surveillance of HIV seroconversions and HIV drug resistance is recommended as new products are introduced, together with optimizing screening and diagnosis of acute HIV acquisition [8].

## 5 LIMITATIONS

It is a notable limitation that blood level testing was not conducted to confirm PrEP use, and was estimated through medication pick‐ups. Although all test kits used had high specificity and sensitivity, the variation in test kits used across sites may have contributed to geographical differences in HIV diagnoses. There was also limited data on clients’ self‐reported PrEP use, perceived vulnerability to HIV and reasons for PrEP interruption. Data on clients who did not return for follow‐up, and who may have seroconverted after discontinuation of PrEP was also not collected; we were unable to calculate a true rate of seroconversion and the seroconversions represented in this case series may be an underestimate of actual seroconversions occurring. There may have been a selection bias, as clients returning for services may have been more able to access and be using PrEP, than those who did not come back.

## 6 CONCLUSIONS

This study confirmed a low number of seroconversions among a large cohort of PrEP users in a real‐world implementation study, the majority of which occurred among clients who had interrupted or discontinued PrEP use. Although probable missed acute HIV acquisitions were low, ongoing research will be needed to inform and optimize HIV testing approaches as long‐acting PrEP methods are introduced.

## COMPETING INTERESTS

The authors have no competing interests to declare.

## AUTHORS’ CONTRIBUTIONS

CEM led the writing of the manuscript. HR, LAC and GC conducted the data cleaning and analysis. SM provided oversight of the Project PrEP study design and implementation. All authors discussed the results and contributed to the writing of the manuscript, review of drafts and approval of the final manuscript.

## FUNDING

This study was funded through Unitaid, grant number 2017‐21‐Wits‐PrEP.

## Data Availability

The data that support the findings of this study are available on request from the corresponding author.
